# Crystal structure of methyl 2-(7-hy­droxy-2-oxo-2*H*-chromen-4-yl)acetate

**DOI:** 10.1107/S2056989015014061

**Published:** 2015-08-22

**Authors:** Sammer Yousuf, Shafqat Hussain, Khalid Mohammed Khan, Muhammad Shabeer, Shahnaz Perveen

**Affiliations:** a H.E.J. Research Institute of Chemistry, International Center for Chemical and Biological Sciences, University of Karachi, Karachi 75270, Pakistan; b Department of Chemistry, Karakoram International University, Gilgit, Pakistan; c PCSIR Laboratories, Karachi, Pakistan

**Keywords:** crystal structure, ester, coumarin, chromene, hydrogen bonding

## Abstract

In the title coumarin derivative, C_12_H_10_O_5_, the fused ring system is almost planar (r.m.s deviation = 0.016 Å). The C_ar_—C—C=O torsion angle of the side chain is −8.4 (2)° In the crystal, mol­ecules are linked by O—H⋯O hydrogen bonds, generating *C*(8) chains propagating in the [100] direction. The chains are cross-linked by weak C—H⋯O inter­actions, thereby generating undulating (001) sheets.

## Related literature   

For the applications and biological activities of coumarin derivatives, see: Vukovic *et al.* (2010 ▸); Basanagouda *et al.* (2009 ▸); Ahmad *et al.* (2008 ▸); Abd Elhafez *et al.* (2003 ▸); Ukhov *et al.* (2001 ▸); Emmanuel-Giota *et al.* (2001 ▸). For the crystal structure of a related compound, see: Subramanian *et al.* (1990 ▸).
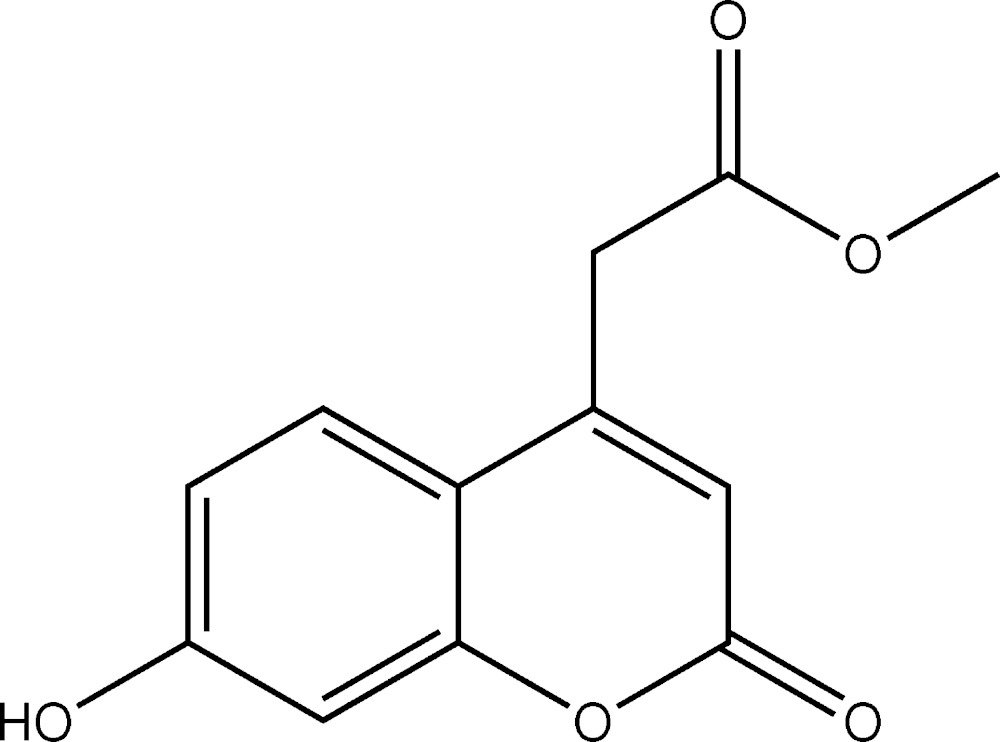


## Experimental   

### Crystal data  

C_12_H_10_O_5_
*M* *_r_* = 234.20
Orthorhombic, 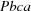

*a* = 13.0780 (12) Å
*b* = 7.2354 (7) Å
*c* = 22.262 (2) Å
*V* = 2106.5 (3) Å^3^
*Z* = 8
Mo *K*α radiation
μ = 0.12 mm^−1^
*T* = 273 K
0.62 × 0.35 × 0.07 mm

### Data collection  

Bruker SMART APEX CCD diffractometer
Absorption correction: multi-scan (*SADABS*; Bruker, 2000 ▸) *T*_min_ = 0.932, *T*_max_ = 0.992
11536 measured reflections
1958 independent reflections
1669 reflections with *I* > 2σ(*I*)
*R*_int_ = 0.021

### Refinement  

*R*[*F* ^2^ > 2σ(*F* ^2^)] = 0.037
*wR*(*F* ^2^) = 0.104
*S* = 1.03
1958 reflections
158 parameters
H atoms treated by a mixture of independent and constrained refinement
Δρ_max_ = 0.28 e Å^−3^
Δρ_min_ = −0.19 e Å^−3^

### 

Data collection: *SMART* (Bruker, 2000 ▸); cell refinement: *SAINT* (Bruker, 2000 ▸); data reduction: *SAINT*; program(s) used to solve structure: *SHELXS97* (Sheldrick, 2008 ▸); program(s) used to refine structure: *SHELXL97* (Sheldrick, 2008 ▸); molecular graphics: *SHELXTL* (Sheldrick, 2008 ▸); software used to prepare material for publication: *PLATON* (Spek, 2009 ▸).

## Supplementary Material

Crystal structure: contains datablock(s) global, I. DOI: 10.1107/S2056989015014061/hb7469sup1.cif

Structure factors: contains datablock(s) I. DOI: 10.1107/S2056989015014061/hb7469Isup2.hkl

Supporting information file. DOI: 10.1107/S2056989015014061/hb7469Isup3.cml

**Fig. 1.**
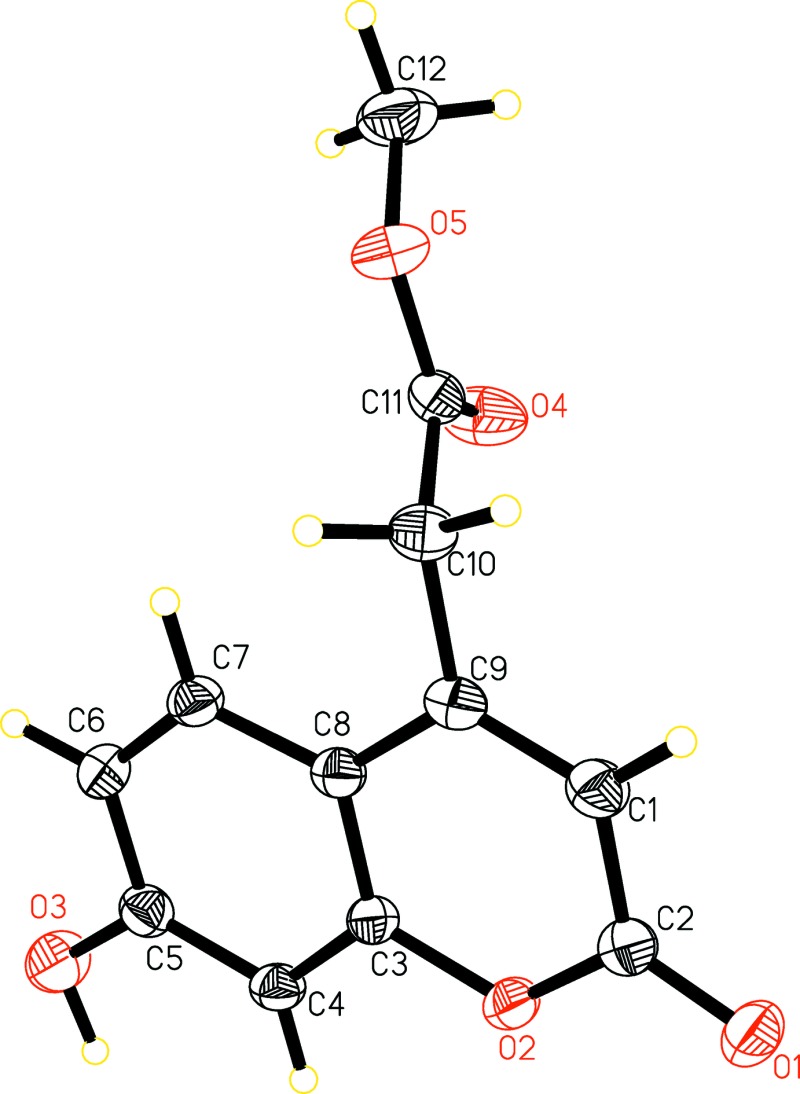
The molecular structure of (I) with displacement ellipsoids drawn at 30% probability level.

. DOI: 10.1107/S2056989015014061/hb7469fig1.tif
The mol­ecular structure of (I) with displacement ellipsoids drawn at 30% probability level.

**Fig. 2.**
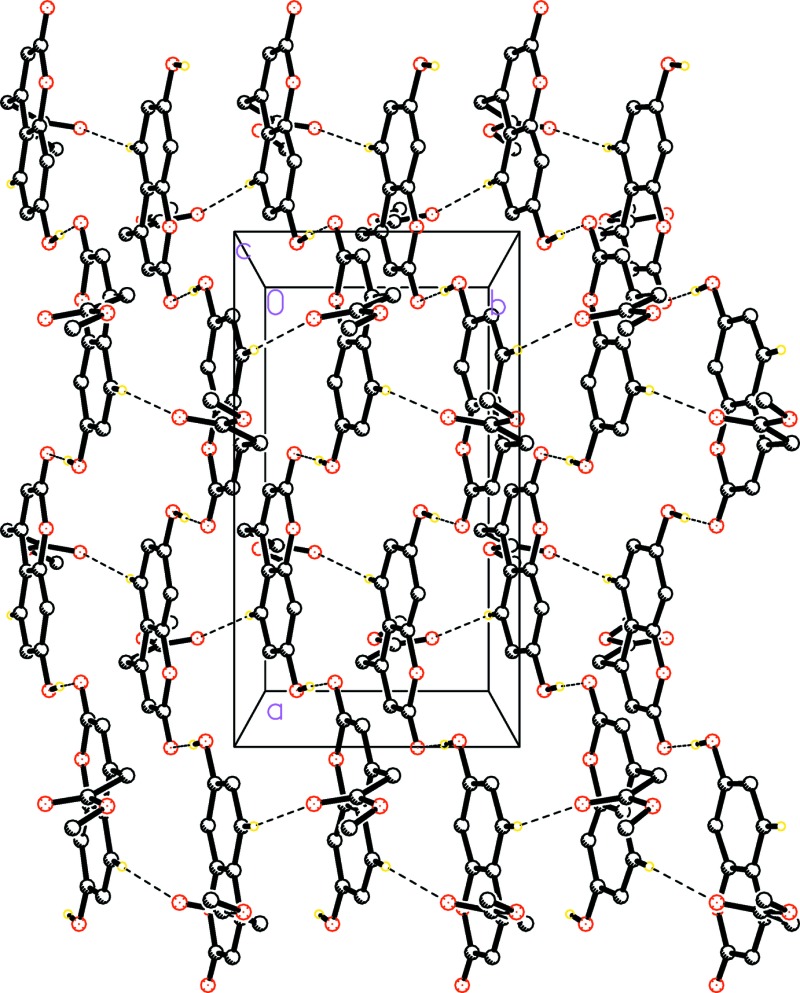
The crystal packing of the title compound I. Only hydrogen atoms involved in hydrogen bonding are shown.

. DOI: 10.1107/S2056989015014061/hb7469fig2.tif
The crystal packing of the title compound I. Only hydrogen atoms involved in hydrogen bonding are shown.

CCDC reference: 1415274

Additional supporting information:  crystallographic information; 3D view; checkCIF report

## Figures and Tables

**Table 1:** Hydrogen-bond geometry (, )

*D*H*A*	*D*H	H*A*	*D* *A*	*D*H*A*
O3H3O1^i^	0.96(2)	1.74(2)	2.7002(17)	177(2)
C7H7*A*O4^ii^	0.93	2.47	3.3320(18)	155

Symmetry codes: (i) 
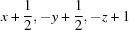
; (ii) 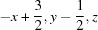.

## supplementary crystallographic information

### S1. Comment

Coumarin, 2*H*-chromen-2-ones are naturally occurring aroma containing organic molecules belongs to benzopyrone (Ahmad *et al.*, 2008) class of compounds. Coumarines known to have wide range of biological activities including antibacterial (Abd Elhafez *et al.*, 2003, Ukhov *et al.*, 2001), antitumour and anticoagulant (Emmanuel-Giota *et al.*, 2001), antioxidant (Basanagouda *et al.*, 2009) and antiinflammatory (Vukovic *et al.*, 2010) properties. The literature has disclosed various methodologies to synthesize coumarine and their structural analogues. The title compound was synthesized during our attempts to maintained libraries of structural analogues of bioactive organic molecules.

The structure of title compound is similar to that of previously published Ethyl 7-hydroxy-4-coumarinacetate (Subramanian *et al.*, 1990) with the difference that ethyl acetate moiety is replaced by methyl acetate chain (O4—O5/C10–C12)attached at C9 of central coumarin ring (Fig. 1).

The crystal structure features O3—H3···O1, and C7—H7A···O4 interactions to form (001) sheets (symmetry codes as in Table 2 and Fig. 2).

### S2. Experimental

2-(7-Hydroxy-2-oxo-2*H*-chromen-4-yl) acetic acid (220 mg, 1 mmol) was dissolved in methanol (15 ml), and a few drops of sulfuric acid were added. The resulting reaction mixture was refluxed for 3 h. After the completion of the reaction as indicated by TLC, solvent was evaporated and the resulting reaction mixture was extracted with ethyl acetate, washed with sodium bicarbonate, brine and dried over anhydrous sodium sulfate. The solvent was removed under reduced pressure to afford crystals of title compound in 215 mg, 91% yield.

### S3. Refinement

H atoms on methyl, methylene and phenyl were positioned geometrically with C—H = 0.96 Å (CH_3_), 0.97 Å (CH_2_) and 0.93 Å (CH phenyl) and constrained to ride on their parent atoms with *U*_iso_(H)=1.5*U*_eq_(CH_3_) and 1.2*U*_eq_(CH and CH_2_). The H atoms on the oxygen (O–H= 0.96 (2) Å) was located in difference Fourier map and refined isotropically.

### Crystal data

**Table:**

C_12_H_10_O_5_	*D*_x_ = 1.477 Mg m^−^^3^
*M**_r_* = 234.20	Mo *K*α radiation, λ = 0.71073 Å
Orthorhombic, *P**b**c**a*	Cell parameters from 4477 reflections
*a* = 13.0780 (12) Å	θ = 2.4–28.3°
*b* = 7.2354 (7) Å	µ = 0.12 mm^−^^1^
*c* = 22.262 (2) Å	*T* = 273 K
*V* = 2106.5 (3) Å^3^	Plate, yellow
*Z* = 8	0.62 × 0.35 × 0.07 mm
*F*(000) = 976	

### Data collection

**Table:**

Bruker SMART APEX CCD diffractometer	1958 independent reflections
Radiation source: fine-focus sealed tube	1669 reflections with *I* > 2σ(*I*)
Graphite monochromator	*R*_int_ = 0.021
ω scan	θ_max_ = 25.5°, θ_min_ = 1.8°
Absorption correction: multi-scan (*SADABS*; Bruker, 2000)	*h* = −15→14
*T*_min_ = 0.932, *T*_max_ = 0.992	*k* = −8→8
11536 measured reflections	*l* = −26→26

### Refinement

**Table:**

Refinement on *F*^2^	Primary atom site location: structure-invariant direct methods
Least-squares matrix: full	Secondary atom site location: difference Fourier map
*R*[*F*^2^ > 2σ(*F*^2^)] = 0.037	Hydrogen site location: inferred from neighbouring sites
*wR*(*F*^2^) = 0.104	H atoms treated by a mixture of independent and constrained refinement
*S* = 1.03	*w* = 1/[σ^2^(*F*_o_^2^) + (0.0523*P*)^2^ + 0.6817*P*] where *P* = (*F*_o_^2^ + 2*F*_c_^2^)/3
1958 reflections	(Δ/σ)_max_ < 0.001
158 parameters	Δρ_max_ = 0.28 e Å^−^^3^
0 restraints	Δρ_min_ = −0.19 e Å^−^^3^

### Special details

**Table:**

Geometry. All e.s.d.'s (except the e.s.d. in the dihedral angle between two l.s. planes) are estimated using the full covariance matrix. The cell e.s.d.'s are taken into account individually in the estimation of e.s.d.'s in distances, angles and torsion angles; correlations between e.s.d.'s in cell parameters are only used when they are defined by crystal symmetry. An approximate (isotropic) treatment of cell e.s.d.'s is used for estimating e.s.d.'s involving l.s. planes.
Refinement. Refinement of *F*^2^ against ALL reflections. The weighted *R*-factor *wR* and goodness of fit *S* are based on *F*^2^, conventional *R*-factors *R* are based on *F*, with *F* set to zero for negative *F*^2^. The threshold expression of *F*^2^ > σ(*F*^2^) is used only for calculating *R*-factors(gt) *etc*. and is not relevant to the choice of reflections for refinement. *R*-factors based on *F*^2^ are statistically about twice as large as those based on *F*, and *R*- factors based on ALL data will be even larger.

### Fractional atomic coordinates and isotropic or equivalent isotropic displacement parameters (Å^2^)

**Table:**

	*x*	*y*	*z*	*U*_iso_*/*U*_eq_	
O1	0.42044 (8)	0.1647 (2)	0.43882 (5)	0.0591 (4)	
O2	0.58807 (7)	0.16062 (15)	0.44081 (4)	0.0403 (3)	
O3	0.94960 (9)	0.1761 (2)	0.45283 (6)	0.0597 (4)	
O4	0.64831 (11)	0.23456 (17)	0.22259 (5)	0.0633 (4)	
O5	0.64257 (9)	−0.01095 (16)	0.16271 (5)	0.0525 (3)	
C1	0.50365 (12)	0.0564 (2)	0.35141 (6)	0.0440 (4)	
H1A	0.4432	0.0350	0.3305	0.053*	
C2	0.49845 (11)	0.1288 (2)	0.41145 (7)	0.0432 (4)	
C3	0.68119 (11)	0.12502 (18)	0.41444 (6)	0.0338 (3)	
C4	0.76625 (11)	0.1663 (2)	0.44865 (6)	0.0365 (3)	
H4A	0.7595	0.2141	0.4872	0.044*	
C5	0.86154 (11)	0.1347 (2)	0.42403 (6)	0.0397 (4)	
C6	0.87047 (12)	0.0581 (2)	0.36677 (7)	0.0414 (4)	
H6A	0.9349	0.0341	0.3509	0.050*	
C7	0.78507 (11)	0.01819 (19)	0.33383 (6)	0.0376 (3)	
H7A	0.7923	−0.0328	0.2957	0.045*	
C8	0.68705 (11)	0.05257 (18)	0.35637 (6)	0.0336 (3)	
C9	0.59328 (12)	0.01865 (19)	0.32448 (6)	0.0375 (3)	
C10	0.59598 (12)	−0.0602 (2)	0.26187 (6)	0.0426 (4)	
H10A	0.6403	−0.1677	0.2617	0.051*	
H10B	0.5278	−0.1013	0.2512	0.051*	
C11	0.63285 (11)	0.0736 (2)	0.21485 (6)	0.0379 (3)	
C12	0.67778 (15)	0.1002 (3)	0.11240 (8)	0.0640 (5)	
H12A	0.6822	0.0244	0.0771	0.096*	
H12B	0.7440	0.1503	0.1214	0.096*	
H12C	0.6304	0.1993	0.1054	0.096*	
H3	0.9369 (17)	0.231 (3)	0.4915 (11)	0.091 (7)*	

### Atomic displacement parameters (Å^2^)

**Table:**

	*U*^11^	*U*^22^	*U*^33^	*U*^12^	*U*^13^	*U*^23^
O1	0.0375 (6)	0.0935 (10)	0.0463 (7)	0.0056 (6)	0.0053 (5)	−0.0091 (6)
O2	0.0358 (6)	0.0533 (6)	0.0318 (5)	0.0014 (5)	0.0024 (4)	−0.0043 (4)
O3	0.0379 (7)	0.0923 (10)	0.0489 (7)	−0.0035 (6)	−0.0062 (5)	−0.0117 (6)
O4	0.1002 (11)	0.0475 (7)	0.0424 (6)	−0.0102 (7)	−0.0047 (6)	−0.0019 (5)
O5	0.0647 (8)	0.0582 (7)	0.0345 (6)	−0.0042 (6)	0.0086 (5)	−0.0083 (5)
C1	0.0401 (9)	0.0549 (9)	0.0371 (8)	−0.0024 (7)	−0.0052 (6)	−0.0016 (7)
C2	0.0390 (8)	0.0527 (9)	0.0379 (8)	0.0009 (7)	0.0006 (6)	0.0009 (7)
C3	0.0375 (8)	0.0340 (7)	0.0298 (7)	0.0035 (6)	0.0027 (6)	0.0018 (5)
C4	0.0420 (9)	0.0400 (7)	0.0276 (6)	0.0000 (6)	−0.0010 (6)	−0.0025 (5)
C5	0.0387 (8)	0.0436 (8)	0.0368 (8)	−0.0002 (6)	−0.0041 (6)	0.0017 (6)
C6	0.0391 (8)	0.0451 (8)	0.0401 (8)	0.0053 (7)	0.0056 (6)	−0.0003 (6)
C7	0.0455 (9)	0.0360 (7)	0.0314 (7)	0.0038 (6)	0.0032 (6)	−0.0024 (6)
C8	0.0399 (8)	0.0310 (7)	0.0300 (7)	0.0009 (6)	−0.0003 (6)	−0.0001 (5)
C9	0.0451 (9)	0.0360 (7)	0.0314 (7)	−0.0023 (6)	−0.0024 (6)	0.0007 (6)
C10	0.0486 (9)	0.0446 (8)	0.0345 (8)	−0.0049 (7)	−0.0046 (6)	−0.0055 (6)
C11	0.0346 (8)	0.0464 (8)	0.0327 (7)	0.0005 (6)	−0.0059 (6)	−0.0049 (6)
C12	0.0679 (12)	0.0845 (14)	0.0394 (9)	−0.0071 (10)	0.0132 (8)	0.0016 (9)

### Geometric parameters (Å, º)

**Table:**

O1—C2	1.2165 (18)	C4—H4A	0.9300
O2—C2	1.3617 (18)	C5—C6	1.395 (2)
O2—C3	1.3764 (17)	C6—C7	1.367 (2)
O3—C5	1.3515 (18)	C6—H6A	0.9300
O3—H3	0.96 (2)	C7—C8	1.399 (2)
O4—C11	1.1944 (19)	C7—H7A	0.9300
O5—C11	1.3183 (17)	C8—C9	1.438 (2)
O5—C12	1.454 (2)	C9—C10	1.5064 (19)
C1—C9	1.345 (2)	C10—C11	1.505 (2)
C1—C2	1.437 (2)	C10—H10A	0.9700
C1—H1A	0.9300	C10—H10B	0.9700
C3—C4	1.381 (2)	C12—H12A	0.9600
C3—C8	1.3971 (19)	C12—H12B	0.9600
C4—C5	1.380 (2)	C12—H12C	0.9600
			
C2—O2—C3	121.67 (11)	C8—C7—H7A	119.4
C5—O3—H3	111.6 (14)	C3—C8—C7	116.67 (12)
C11—O5—C12	116.88 (13)	C3—C8—C9	118.30 (13)
C9—C1—C2	122.00 (13)	C7—C8—C9	125.03 (12)
C9—C1—H1A	119.0	C1—C9—C8	119.25 (13)
C2—C1—H1A	119.0	C1—C9—C10	120.65 (13)
O1—C2—O2	116.43 (13)	C8—C9—C10	120.09 (13)
O1—C2—C1	125.70 (14)	C11—C10—C9	114.04 (12)
O2—C2—C1	117.87 (13)	C11—C10—H10A	108.7
O2—C3—C4	115.91 (12)	C9—C10—H10A	108.7
O2—C3—C8	120.90 (12)	C11—C10—H10B	108.7
C4—C3—C8	123.19 (13)	C9—C10—H10B	108.7
C5—C4—C3	118.19 (13)	H10A—C10—H10B	107.6
C5—C4—H4A	120.9	O4—C11—O5	124.29 (14)
C3—C4—H4A	120.9	O4—C11—C10	125.51 (13)
O3—C5—C4	122.97 (13)	O5—C11—C10	110.16 (13)
O3—C5—C6	116.74 (14)	O5—C12—H12A	109.5
C4—C5—C6	120.28 (13)	O5—C12—H12B	109.5
C7—C6—C5	120.38 (14)	H12A—C12—H12B	109.5
C7—C6—H6A	119.8	O5—C12—H12C	109.5
C5—C6—H6A	119.8	H12A—C12—H12C	109.5
C6—C7—C8	121.25 (13)	H12B—C12—H12C	109.5
C6—C7—H7A	119.4		
			
C3—O2—C2—O1	179.92 (13)	C4—C3—C8—C9	−178.69 (13)
C3—O2—C2—C1	−0.1 (2)	C6—C7—C8—C3	−1.5 (2)
C9—C1—C2—O1	−179.37 (16)	C6—C7—C8—C9	178.62 (13)
C9—C1—C2—O2	0.7 (2)	C2—C1—C9—C8	−0.4 (2)
C2—O2—C3—C4	178.95 (12)	C2—C1—C9—C10	178.94 (14)
C2—O2—C3—C8	−0.7 (2)	C3—C8—C9—C1	−0.4 (2)
O2—C3—C4—C5	−179.42 (12)	C7—C8—C9—C1	179.48 (14)
C8—C3—C4—C5	0.2 (2)	C3—C8—C9—C10	−179.72 (13)
C3—C4—C5—O3	177.69 (14)	C7—C8—C9—C10	0.1 (2)
C3—C4—C5—C6	−1.8 (2)	C1—C9—C10—C11	108.63 (16)
O3—C5—C6—C7	−177.79 (14)	C8—C9—C10—C11	−72.02 (18)
C4—C5—C6—C7	1.8 (2)	C12—O5—C11—O4	2.1 (2)
C5—C6—C7—C8	0.0 (2)	C12—O5—C11—C10	−179.98 (14)
O2—C3—C8—C7	−178.95 (12)	C9—C10—C11—O4	−8.4 (2)
C4—C3—C8—C7	1.5 (2)	C9—C10—C11—O5	173.79 (13)
O2—C3—C8—C9	0.91 (19)		

### Hydrogen-bond geometry (Å, º)

**Table:**

*D*—H···*A*	*D*—H	H···*A*	*D*···*A*	*D*—H···*A*
O3—H3···O1^i^	0.96 (2)	1.74 (2)	2.7002 (17)	177 (2)
C7—H7*A*···O4^ii^	0.93	2.47	3.3320 (18)	155

Symmetry codes: (i) *x*+1/2, −*y*+1/2, −*z*+1; (ii) −*x*+3/2, *y*−1/2, *z*.

## References

[bb4] Abd Elhafez, O. M., El Khrisy, E. E. D. A. M., Badria, F. & Fathy, A. E. D. M. (2003). *Arch. Pharm. Res.* **26**, 686–696. 10.1007/BF02976675 14560914

[bb1] Ahmad, H. B., Malana, M. A., Rama, N. H., Ilyas, S., Yousuf, M. & Khan, K. M. (2008). *J. Chem. Soc. Pakistan*, **30**, 834–844.

[bb2] Basanagouda, M., Kulkarni, M. V., Sharma, D., Gupta, V. K., Pranesha, Sandhyarani, P. & Rasal, V. P. (2009). *J. Chem. Sci.* **121**, 485–495.

[bb3] Bruker (2000). *SADABS*, *SMART* and *SAINT*. Bruker AXS Inc., Madison, Wisconsin, USA.

[bb5] Emmanuel-Giota, A. A., Fylaktakidou, K. C., Litinas, K. E., Nicolaides, D. N. & Hadjipavlou-Litina, D. J. (2001). *J. Heterocycl. Chem.* **38**, 717–722.

[bb6] Sheldrick, G. M. (2008). *Acta Cryst.* A**64**, 112–122. 10.1107/S0108767307043930 18156677

[bb7] Spek, A. L. (2009). *Acta Cryst.* D**65**, 148–155. 10.1107/S090744490804362X PMC2631630 19171970

[bb8] Subramanian, K., Sivakumar, K., Natarajan, S. & Parthasarathy, S. (1990). *Acta Cryst* C**46**, 1661–1663.

[bb9] Ukhov, S. V., Kon’shin, M. E. & Odegova, T. F. (2001). *Pharm. Chem. J.* **35**, 364–365.

[bb10] Vukovic, N., Sukdolak, S., Solujic, S. & Niciforovic, N. (2010). *Arch. Pharm. Res.* **33**, 5–15. 10.1007/s12272-010-2220-z 20191339

