# Supplementary figures and images for: Crystal structure of methyl 2-(7-hy­droxy-2-oxo-2H-chromen-4-yl)acetate
Source: Acta Crystallogr E Crystallogr Commun. 2015 Aug 22;71(Pt 9):o677–8. doi: 10.1107/S2056989015014061 (PMC4555392; doi:10.1107/S2056989015014061)

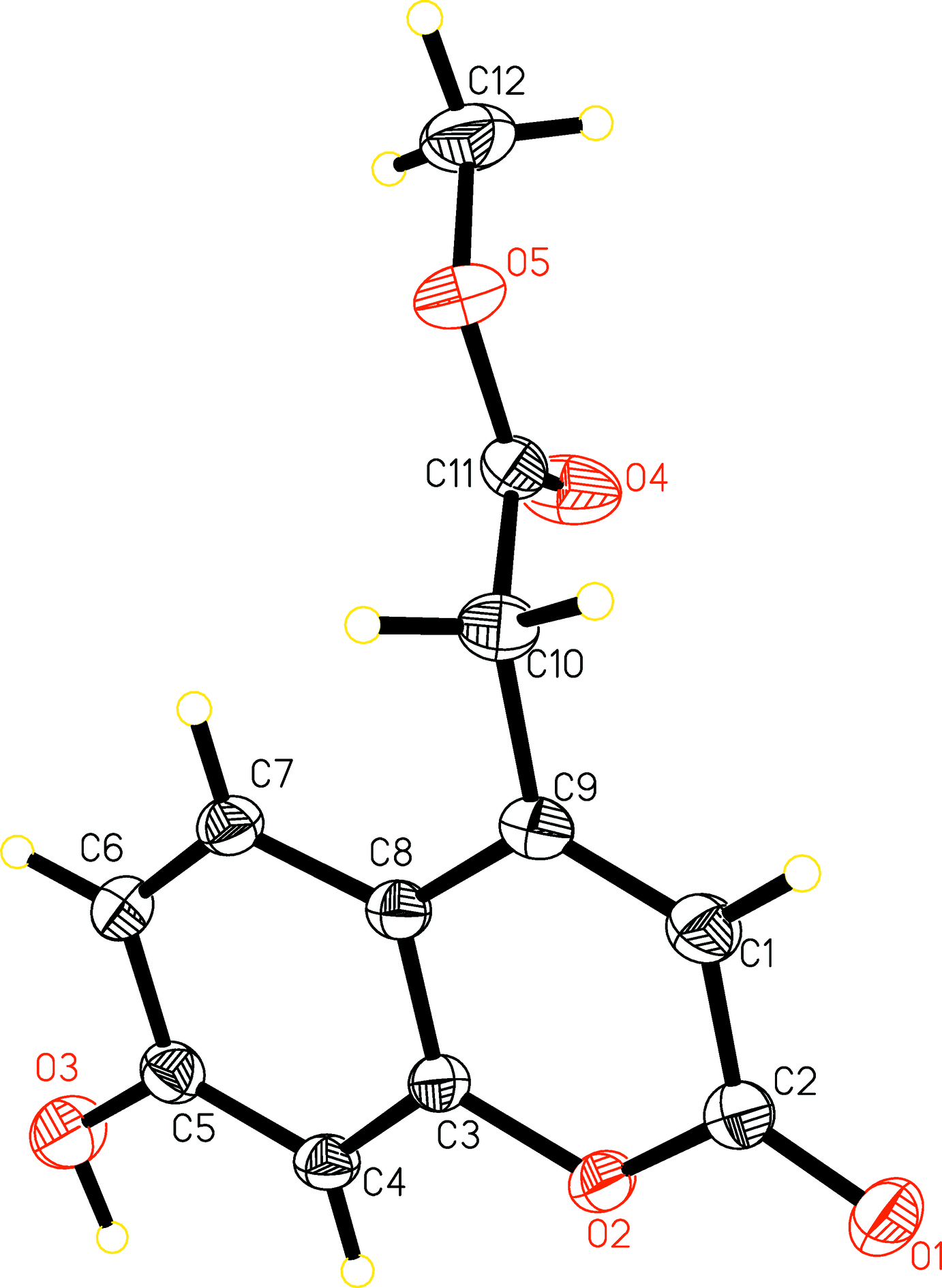

Supplement: Supplementary file 4 [file e-71-0o677-fig1.tif]

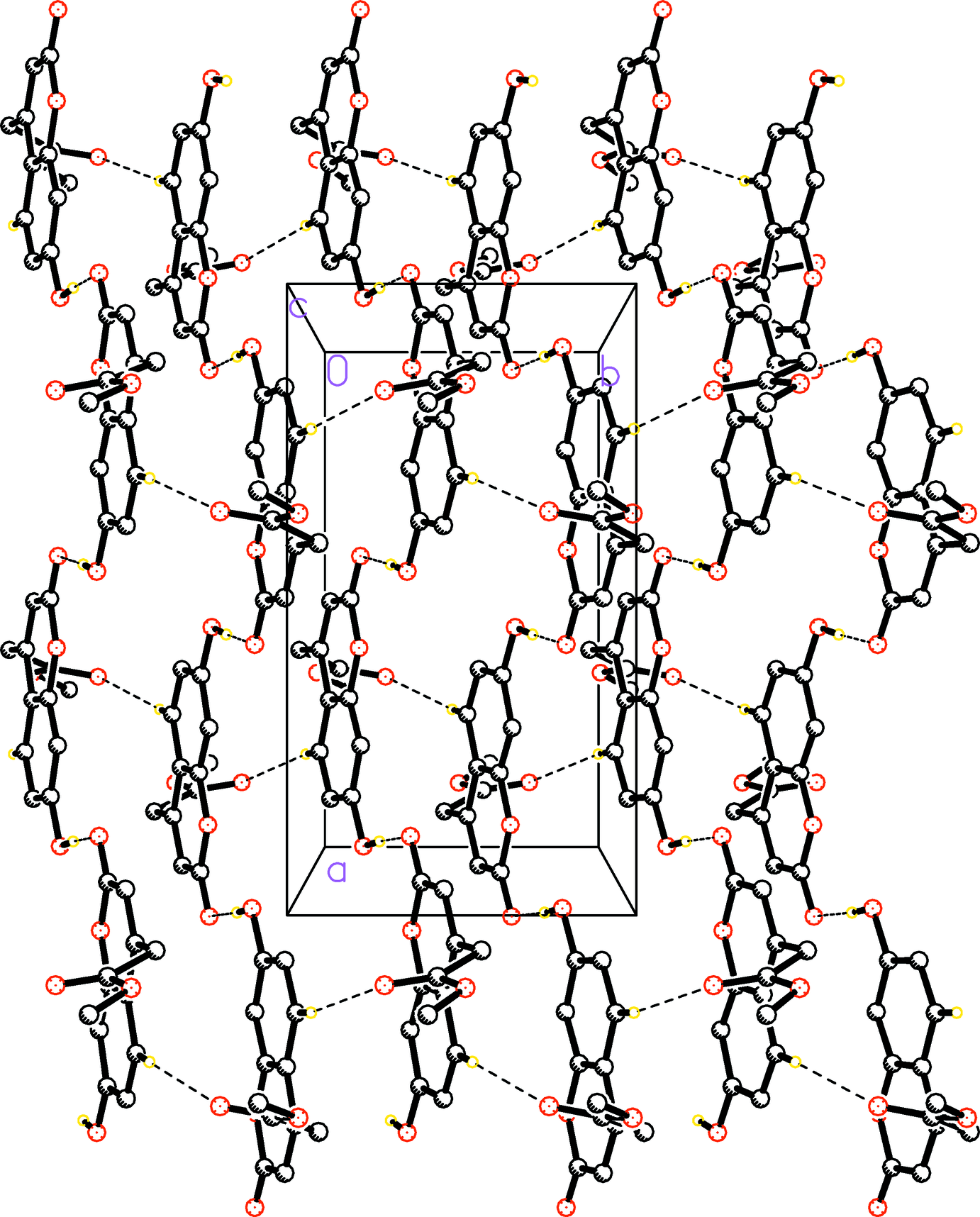

Supplement: Supplementary file 5 [file e-71-0o677-fig2.tif]
